# Improving district level health planning and priority setting in Tanzania through implementing accountability for reasonableness framework: Perceptions of stakeholders

**DOI:** 10.1186/1472-6963-10-322

**Published:** 2010-12-01

**Authors:** Stephen Maluka, Peter Kamuzora, Miguel San Sebastián, Jens Byskov, Benedict Ndawi, Anna-Karin Hurtig

**Affiliations:** 1 Institute of Development Studies, University of Dar Es Salaam, P.O. Box 35169 Dar Es Salaam, Tanzania; 2 Umeå International School of Public Health (UISPH), Umea University SE 90185 Umeå, Sweden; 3 DBL - Centre for Health Research and Development, Faculty of Life Sciences, University of Copenhagen, Thorvaldsensvej 57, DK 1871 Frederiksberg, Denmark; 4 Primary Health Care Institute (PHCI), P.O. Box 235, Iringa, Tanzania

## Abstract

**Background:**

In 2006, researchers and decision-makers launched a five-year project - Response to Accountable Priority Setting for Trust in Health Systems (REACT) - to improve planning and priority-setting through implementing the Accountability for Reasonableness framework in Mbarali District, Tanzania. The objective of this paper is to explore the acceptability of Accountability for Reasonableness from the perspectives of the Council Health Management Team, local government officials, health workforce and members of user boards and committees.

**Methods:**

Individual interviews were carried out with different categories of actors and stakeholders in the district. The interview guide consisted of a series of questions, asking respondents to describe their perceptions regarding each condition of the Accountability for Reasonableness framework in terms of priority setting. Interviews were analysed using thematic framework analysis. Documentary data were used to support, verify and highlight the key issues that emerged.

**Results:**

Almost all stakeholders viewed Accountability for Reasonableness as an important and feasible approach for improving priority-setting and health service delivery in their context. However, a few aspects of Accountability for Reasonableness were seen as too difficult to implement given the socio-political conditions and traditions in Tanzania. Respondents mentioned: budget ceilings and guidelines, low level of public awareness, unreliable and untimely funding, as well as the limited capacity of the district to generate local resources as the major contextual factors that hampered the full implementation of the framework in their context.

**Conclusion:**

This study was one of the first assessments of the applicability of Accountability for Reasonableness in health care priority-setting in Tanzania. The analysis, overall, suggests that the Accountability for Reasonableness framework could be an important tool for improving priority-setting processes in the contexts of resource-poor settings. However, the full implementation of Accountability for Reasonableness would require a proper capacity-building plan, involving all relevant stakeholders, particularly members of the community since public accountability is the ultimate aim, and it is the community that will live with the consequences of priority-setting decisions.

## Background

Because no health system can afford to provide all possible services and treatments for the people it serves, each system must set priorities regarding what it will, and what it will not, provide. Priority-setting entails identifying systematic rules to decide on the distribution of limited health care resources among competing programmes or patients. It occurs at all levels of every health care system, and is one of the most important issues in health care management today [1,2]. Two key issues lie at the heart of setting priorities, namely: legitimacy and fairness. The legitimacy question asks: why, and under what conditions, should authority over priority-setting be placed in the hands of a particular organisation, group or person? The fairness question asks: when should users and providers of services (a patient or clinician) accept a particular priority-setting decision as fair? [3]. Fundamentally, priority setting involves choices about values, among which evidence is important, but not sufficient. However, values often conflict and people disagree about which values should dominate [4].

In the absence of consensus about which values should guide the priority-setting process, Accountability for Reasonableness (A4R) was developed based on identification of main features of a number of the best-performing health care organisations, and has been suggested as an important tool for putting in place procedures that will ensure fairness and legitimacy of the prioritisation process [1,5-7]. A4R is a comprehensive framework which provides structure for stakeholders to establish priorities for their specific contexts, while taking into account limited resources and regulatory conditions. The A4R framework consists of four conditions:

▪ Relevance to the local setting as decided by agreed criteria.

▪ Publicising priority-setting decisions and the reasons behind them.

▪ Establishment of revision/appeals mechanisms for challenging and revising decisions (in the light of additional evidence and values).

▪ Provision of leadership to ensure that the first three conditions are met.

A4R has been recognised as an important advance among decision-makers, health care professionals, and scholars involved in empirical studies of priority setting. Developed in the contexts of managed care reform in the United States, the framework has been validated in the Canadian public health system [8-11] and in several other countries [7].

Can this approach to priority-setting apply in low-income countries with the scarcest resources and relatively weak organisations and democratic institutions? In 2006, African researchers, in collaboration with colleagues from Europe, launched the five-year project: Response to Accountable Priority Setting for Trust in Health Systems (REACT). REACT aims at improving priority-setting in health care institutions through implementing the A4R framework in Mbarali District in Tanzania, Malindi District in Kenya and the Kapiri Mposhi District in Zambia [12]. A few empirical studies have used A4R as a conceptual framework to evaluate priority-setting and decision making processes in such settings [13-17], and they have shown that A4R can provide useful guidance. Similarly, another study has recently compared the elements of fairness described in the A4R framework to the elements of fairness as perceived by decision-makers [18]. However, with the notable exception of a study of district health planners in Tanzania [19], there are no studies about the perceptions of stakeholders regarding Accountability for Reasonableness in low-income countries' health care institutions. To date, no study has monitored and evaluated the implementation of the A4R framework in low-income countries. There is, therefore, little understanding of what implementing A4R actually entails. Against this background, this paper explores the acceptability of the A4R framework from the perspectives of district health managers, local government officials, the health workforce, and members of user boards and committees in Mbarali District, Tanzania.

## Methods

### The Study Setting

**Figure 1:**
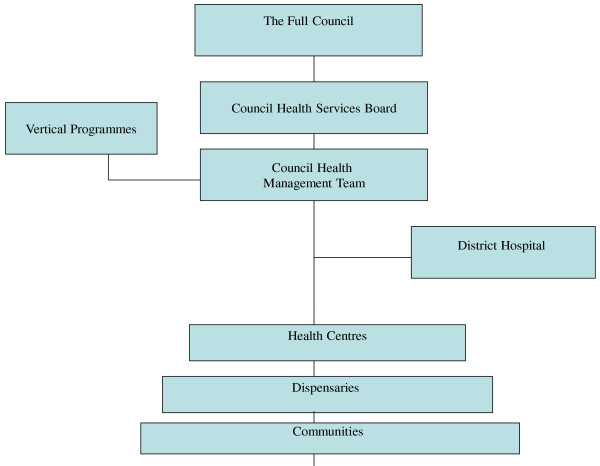
**The structure of district health system in Tanzania**.

The study was conducted in Mbarali District in the Mbeya region of Tanzania. Mbarali District was selected by the REACT project as it was a typical rural district in Tanzania. Mbarali district has two divisions with 11 wards, 98 registered villages, 652 hamlets, and 55,374 households. Based on the 2002 National Population Census, the district had 234,101 people, comprised of 114,738 males and 119,363 females, with an annual growth rate of 2.8 percent. Like other districts in Tanzania, the structure of the health system in Mbarali District has been decentralised (Figure 1). At the district level, the Council Health Management Team (CHMT) was formed with the remit of planning and budgeting for activities needed to manage, control, coordinate and support all health services in the district on a year-to-year basis [20]. District health priorities are integrated in the Comprehensive Council Health Plan (CCHP), which has to make the best use of limited resources in meeting local needs. Other responsibilities of the CHMT include: ensuring implementation of health activities by hospitals, health centres, dispensaries, and communities; and to monitor and evaluate implementation of health activities in the district. The CHMT consists of: the District Medical Officer (chairperson), District Nursing Officer, District Laboratory Technician, District Health Officer, District Pharmacist, District Dental Officer, and District Health Secretary (secretary to the team). Other co-opted members of the CHMT may include: Reproductive and Child Health Coordinator, Tuberculosis and Leprosy Coordinator, Malaria Focal Person, HIV/AIDS Coordinator, and Cold Chain Operator - all of whom may be invited to CHMT meetings as and when the need arises.

The CCHP must be approved by the Council Health Service Board (CHSB), and the final plan is approved at the Full Council Meeting. The Full Council is the highest political body in the district and has overall authority for all district health services. After approval by the Full Council, the CCHP is forwarded to the Ministry of Health and Social Welfare (MoHSW) and the Prime Minister's Office Regional Administration and Local Government (PMO-RALG) for final approval before funds can be disbursed to the Local Government Authority. The CHMT is linked to the communities through user committees and boards, which have been formed in each health centre and dispensary.

### The REACT project in Mbarali District

The REACT research process involves the application of A4R, a scientific assessment of the intervention process as well as an evaluation of the applicability of its conditions to priority setting and the subsequent effects on health systems [12]. A preliminary phase of the implementation of the A4R framework in the district began in 2006, involving gathering baseline data, consultation and planning. The full application of A4R began in 2008, and the project will end in December 2010. The application of A4R includes: describing priority-setting in the district, evaluating the description using A4R, and implementing improvement strategies in a continuous process to address gaps in A4R conditions [21].

The A4R process in Mbarali District is being carried out by the CHMT with support from an Action Research Team (ART). The role of the CHMT is to ensure the application of A4R conditions during the annual planning and priority-setting at the district level, and in day-to-day decision-making processes that concern choice of options within resource limits. The action research is carried out by the ART with support from the rest of the research team members. The relevant results from the baseline and monitoring are communicated to the CHMT through the ART. The ART comprises four members of the CHMT and two researchers from research and academic institutions. The ART team holds meetings once every two months to discuss and review the implementation of A4R in the district. ART members collaborate with other actors to ensure effective implementation of A4R through meetings, sensitisation workshops, informal encounters, and non-participant observations. Stakeholders who have been sensitised about A4R conditions include: Regional Health Management Team (RHMT), Regional Secretariat, District Health Forum (heads of health facilities), councillors (political leaders), Chairperson of Health Facility Governing Committees, non-governmental organizations (NGOs), faith-based organizations (FBOs), community-based organizations (CBOs), heads of Department and the media.

However, while different stakeholders in the district have been sensitised about the A4R framework, the application of A4R has been predominantly focused within the CHMT and at the district hospital, to benefit this level as well as to create an environment conducive to a more comprehensive application of A4R. Though responsiveness to stakeholder and community preferences is inherent in A4R, little effort has, as yet, been made to extend the awareness and application of A4R at the community level.

### Data collection techniques

This paper is primarily based on the individual interviews with key stakeholders in Mbarali District. Documentary data was used to support, verify and highlight the key issues that emerged. Individual interviews were carried out between January and February 2010, almost two years after the beginning of the A4R intervention in the district. Interviews lasted approximately 45 minutes and were carried out at the respondent's workplace and/or home. An interview guide was developed to assist the semi-structured interviews with key respondents (Appendix 1). The interview guide consisted of a series of questions asking respondents to describe their perceptions regarding the applicability and feasibility of each condition of the A4R framework in relation to priority setting. Consistent with qualitative research methods, an open stance was maintained, probing into emerging themes and seeking clarification when necessary. In order to cover a wide range of views of the different actors involved, a purposive sampling technique was used. At the district level, members of the CHMT and Council Health Services Board (CHSB) were interviewed. At the health facility level, committee members at the district hospital and health centres were interviewed. In total, 20 interviews were carried out (see Table 1).

**Table 1:** Data sources

	Source of data	Quantity of data
1	20 Individual Interviews	▪ Seven members of the CHMT
		▪ Two local government officials
		▪ Three members of user committees and boards
		▪ One member of an NGO (advocacy group)
		▪ Two heads of a health facility (health centres)
		▪ Five health workers at the district hospital
2	Documents	▪ Nine minutes of the ART
		▪ Three minutes of the ART/CHMT
		▪ Ten Monthly Observation Reports
		▪ Two observation reports from the planning meetings
		▪ One sensitisation report

To supplement the interviews, considerable documentary information was obtained and our analysis was validated with non-participant observation. Throughout the implementation of the REACT, one researcher participated in the priority-setting exercise. Participant observation notes were taken during all priority-setting meetings and sensitisation workshops. The researchers also documented events related to the implementation of A4R in the district and produced monthly reports. The monthly reports also captured the reactions of different stakeholders on the implementation of the A4R framework in the district. Other documents analysed included minutes of the ART and ART/CHMT meetings, and reports from the sensitisation meetings.

### Data analysis

**Figure 2:**
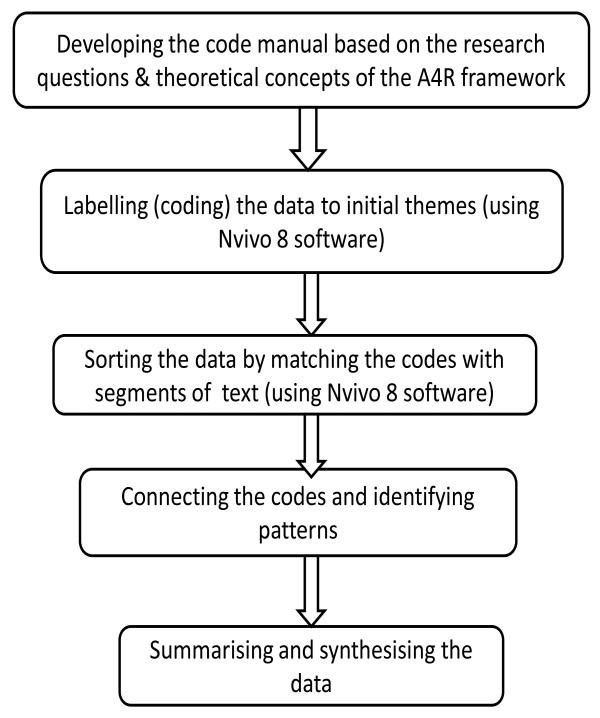
**Analytical steps adopted in the data analysis**.

This study adopted the thematic framework approach, in which data were classified and organised according to key themes, concepts and emergent patterns [22]. The thematic framework analysis involved a series of analytical steps (Figure 2). Although presented as a linear, step-by-step procedure, the research analysis was an iterative and reflexive process: first, the code manual was developed by the first author, based on the research questions and theoretical concepts of the A4R framework. Second, the transcripts of each interview were read through by the first author, in collaboration with two co-authors, and responses were identified to the main questions raised by the study. Using Nvivo 8 software, data were coded to initial themes. Thereafter, data were sorted and grouped together under patterns that were more precise, complete, and generalisable [23]. As patterns of meaning emerged, those that had similarities and differences were identified. Finally, data were summarised and synthesised retaining, as much as possible, key terms, phrases and expressions of the respondents. After this analysis, data were triangulated to allow comparison across sources and different categories of stakeholders. The careful and systematic process of analysis and reflection served to ensure analytical rigour [24]. Finally, all research activities were carefully documented to permit a critical appraisal of the methods.

### Ethical Issues

The research was approved by the University of Dar es Salaam. The clearance was presented to the regional and district authorities, which approved the study in their respective areas. Oral informed consent was obtained from all study participants, who were informed of their right to withdraw from the study at any time they wished without penalty. All the interviews were voice recorded with the permission of participants, and the resulting recordings and transcripts were kept confidential.

## Results

This section primarily presents the findings from the analysis of individual interviews with key stakeholders in Mbarali District. Documentary data were used to support, verify and highlight the key issues that emerged. The findings have been organised according to the four conditions of the A4R framework. Verbatim quotes from the interviews with participants have been included to illustrate the main messages communicated.

### Relevance

According to the relevance condition of A4R, rationales for priority-setting decisions should aim to provide a reasonable explanation of why they were taken. Specifically, a rationale is reasonable if it is based on evidence, other values, reasons or principles accepted by the stakeholders; closely linked to this condition is the inclusion of a broad range of stakeholders in the decision-making process.

#### Broadening values and criteria guiding the priority-setting process: easier said than done?

The planning and priority-setting process in Mbarali District is guided, at least theoretically, by the guidelines and budget ceilings imposed by the Ministry of Health and Social Welfare. The planning guidelines require that district health priorities be identified based on locally available epidemiological data and health service statistics in the light of a nationally defined Essential Health Package (EHP). The guidelines further require that interventions in each priority area (diseases and programme) be selected on the basis of severity, feasibility, and control at cost - these criteria have been adopted by the central government without any significant challenges from stakeholders.

There have been disagreements among CHMT members regarding the relevance of the criteria and principles which have been determined by the central government in their respective contexts. Few members of the CHMT fully accepted the use of *burden of disease *and *cost effectiveness analysis *as stipulated in the Tanzania Essential Health Package. They pointed out that while most of the national priorities were relevant in their own contexts, there was insufficient information available to make evidence-based decisions, and that more effort should be made to ensure the availability of more adequate information. The CHMT members mentioned increased budget and political commitment as ways to improve the availability and use of data.

By contrast, some members of the CHMT acknowledged the importance of broadening the criteria and values guiding the priority-setting process. CHMT members stated that the approaches of burden of disease and cost-effectiveness had to be used together with other relevant values such as *equity *and *trust*. It was also highlighted that some local diseases did not form a part of the Essential Health Package. A few CHMT members also felt that more 'bottom-up' planning from the community level was paramount in bringing local priorities into the District Health Plan.

However, while the members of the CHMT recognised the importance of broadening criteria and values, they expressed their concerns about its feasibility in their own contexts. A majority of the CHMT members felt that adding other values and criteria was complex and easier said than done. They pointed out that, in the vast majority of situations, the criteria to be used in determining priorities had already been set by the Ministry of Health and Social Welfare. Thus, the CHMT members argued that it was difficult to add other criteria. Some members of the CHMT remarked:

"*Even though we identify our own district priorities, at the end of the day we must observe what the planning guidelines say*" (interview with a member of the CHMT).

"*While the guidelines are somehow flexible, there are aspects which allow no room and you are obliged to stick to what the instructions from higher levels demand*" (interview with a member of the CHMT).

The guidelines require that the actual allocation of resources has to be based on budget ceilings, as specified in the National Basket Grant Guidelines. Ceilings are set as a percentage of the total basket grant, and are as follows: the office of the District Medical Officer (15%-20%), district hospital (25%-35%), health centre (15%-20%), dispensaries (15%-20%), voluntary agency hospitals (10%-15%), and community initiatives (5%-10%). Almost all CHMT members believed that a lot of money was allotted to priorities that were not very critical in their district, while priorities that were of great importance to the district got insufficient funding.

#### Stakeholder involvement in priority setting: a persistent challenge?

Analysis of the Monthly Observation Reports, and minutes of the ART and ART/CHMT meetings revealed that almost all CHMT members appreciated the importance of broadening stakeholder involvement in the priority-setting process. Likewise, the analysis of sensitisation reports indicated that almost all stakeholders who were sensitised considered stakeholder participation as an important aspect in ensuring that priorities reflected real needs and demands of the community. Interview data from all categories of respondents were consistent with the documentary data on this point. All respondents were of the opinion that involving multiple stakeholders would ensure consideration of diverse reasoning styles. Many respondents stressed the importance of involving the public in the priority-setting process.

However, a few members of the CHMT raised the concern that most community members were not motivated to take part in the priority-setting process. They pointed out that the community was used to a top-down approach in which leaders decided for them, and that its role was just to implement decisions taken by the CHMT. So, community members were surprised to hear that they should take part in decision making. A few members of the CHMT also said that many members of the community did not have the knowledge, skills and experience to effectively contribute to priority-setting decisions. One respondent expressed:

"*I think the issue of involving other stakeholders is very good. But the main problem is that many of the community members have very low understanding of the priority-setting process...... It needs a long time to educate the public so that they are able to participate effectively*" (interview with a member of the CHMT).

In contrast, members of the user committees and boards were of the opinion that the community knows quite well the problems it faces. They argued that if the CHMT had given the community the opportunity to identify priorities then it would have done it better; as one member of the board illustrated:

"*I think it is important for a board member from the community to participate in the planning meetings. The experts sometimes exclude certain things which are important. Those of us who come from the community know a lot of things, perhaps better than the experts do. Some experts come from distant places and don't know the problems facing our villages. Since we were born and grew up in these villages we know a lot of things and better than the experts do*" (interview with a member of the CHSB).

Similarly, some members of the CHMT confirmed that the public was able to identify priorities. They pointed out that, if facilitated, community members could participate effectively in identifying priorities. One respondent expressed it this way:

"*Before starting implementation of REACT even I myself had a feeling that the general citizenry could not set priorities. Frankly, after starting to involve the public in the process we have found that if facilitated they have so much ability to analyse and identify priorities. We have also found that even in Tanzania Social Action Fund (TASAF) projects the public are able to plan and implement different projects very well*" (interview with a member of the CHMT).

Further analysis of the interviews indicates that almost all CHMT members felt that involving more stakeholders in the planning would require additional resources, which simply were not there. The CHMT members argued that budget ceilings imposed by the national government (which prohibit the CHMT from spending above their budget allocations) would prevent additional stakeholders from attending the planning meetings. When respondents were finally asked how optimal stakeholder engagement could be achieved in the district, availability of resources was the most crucial factor mentioned:

"*The main problem which I see is the budget. You know most of us Tanzanians have a feeling that if you are called to a meeting you have to be paid an allowance. Many people don't know that the plans are for their good*" (interview with a member of the CHMT).

"*The main problem is budget. For this year (2010) we wanted to sit together with representatives of elderly, women and physically challenged group for at least two days during the preparation of the District Health Plan. But we won't be successful because you cannot stay with these people without giving them anything*" (interview with member of the CHMT).

### Publicising priorities to stakeholders: a remarkable change?

According to the publicity condition of A4R, for priority-setting to be regarded as fair, decisions regarding priorities and their rationales must be publicly accessible. Analysis of interviews revealed that district health priorities had become more accessible to the members of the CHMT and hospital workers since the 2008/2009 planning year. The decisions and priorities were communicated to programme grouping leaders and other hospital staff through the staff meetings. Priorities were also translated into Kiswahili (the native language) and were pinned on the notice board at the district hospital, health facilities and ward offices (See table 2). The staff at the district hospital received information informally, usually by word-of-mouth. Stakeholders were requested to channel their comments through heads of department or the DMO's office. They could also put their comments in the suggestion box at the district hospital. However, the communication did not explain the values, criteria and other reasons behind the decisions.

**Table 2:** Examples of published priorities in 2009/2010 District Health Plan

Priority Area	Intervention	Council Health Basket Grants	Block Grants
Reproductive & Child Health	Maternal cond.	-	260,000
	ANC	16,641,600	-
	Obstetric Care	1,278,400	22,151,800
	Post Natal Care	2,410,000	-
	Family Planning	19,350,900	1,791,000
	IMCI	12,579,900	1,885,000
	Immunization	27,543,100	27,315,000
	Post abortion care	-	-
		**79,803,900**	**53,402,800**

Communicable diseases	Malaria	24,775,100	2,630,000
	TB/Leprosy	5,467,100	4,100,000
	HIV/AIDS/STD	23,108,100	7,636,300
	Epidemics	9,831,600	3,080,000
		**63,181,900**	**17,446,300**

Non communicable diseases	Cardiovascular disease	4,850,000	-
	Diabetes	11,010,400	-
	Injuries/Trauma	-	-
	Mental Health	-	-
		**15,860,400**	-

Other diseases	Other diseases	1,200,000	520,000
	Eye diseases	6,237,000	1,460,000
	Ear diseases	-	-
	Oral conditions	2,534,000	-
		**9,971,000**	**1,980,000**

Health Promotion	Information Education and Communication	250,000	3,897,300
	Water and sanitation	10,170,000	6,730,000
	School health promotion		8,973,100
	Improved housing		10,000,000
	Occ. Health, Safety	-	3,672,000
		**29,393,100**	**14,299,300**

Strengthening organizational structures & institutional capacities	Personal emolument		1, 256,166,640
	Retention of workers	14, 750,000	108,800,000
	Staff productivity	13, 522,000	
	Human resource Information system	21,679,000	5,070,000
		**49,951,600**	**1, 379,636,640**

Further analysis of interview data from all categories of key respondents showed that disseminating priorities and rationales created greater transparency and provided opportunities for stakeholders, including the community, to know the priorities which had been incorporated in the district health plan. The majority of those who were interviewed reported that, by being transparent, the CHMT offered the people an opportunity to follow-up the implementation of various activities in the plan. Interviewees also said that less rumour and distrust arose when people were informed why certain priorities were included in the district health plan and others not. Some key respondents elaborated as follows:

"*.... Priorities are published on the notice boards so that stakeholders can read them. CHMT, too, has realised the necessity of making the priorities known to patients. The patients read them on the notice boards. Sometimes they ask us for clarification. Indeed, many people are happy about this practice and want it to continue*" (interview with a member of the CHMT).

"*Since last year (2009) the hospital's priorities have been published on notice boards. This is a good thing and we wish the practice should be progressive*.... *Publicity is very important because it enables stakeholders to know the district priorities and be able to question when implementation is stuck.... *" (interview with a health worker).

However, a few members of the CHMT pointed out that the convoluted process to approve district health plans, coupled with the delay in the disbursement of funds by the central government, made it difficult to publicise district priorities. They argued that because the priorities included in the plan were supported by budget decisions, it was not possible to release the plan prior to the release of the budget. Therefore, the CHMT members felt that they were put at a disadvantage because there was no firm date for the release of the funds, and, therefore, changes of specific elements that will be required in plans were not known.

### Appeals/revision: a new culture?

According to the *appeals/revision *condition of A4R, a fair priority-setting process must provide mechanisms for challenging resolutions regarding limit-setting decisions and, more broadly, opportunities for revision and improvement of policies in the light of new evidence. During the period when this study was carried out, no formal appeals mechanism had been put in place. Procedurally, in the implementation of the REACT project in the district, the ART members had started with the *relevance *and *publicity *conditions of A4R. Efforts were under the way to introduce the *appeals *condition. However, interview data revealed that a vast majority of CHMT members believed that their involvement in planning and priority-setting had increased over the past two years. The CHMT members reported that they were now able to appeal against solitary DMO decisions. In addition, the CHMT had started initiatives to publicise priorities on the notice boards at the district hospital. CHMT members also had disseminated priorities to twelve villages in the district. Further, the CHMT had written letters to all heads of health centres and dispensaries asking them to disseminate priorities to the people and give them an opportunity to give feedback. Respondents were asked their perceptions about the relevance and feasibility of the appeals/revision condition of the A4R in their context.

Interview data indicated that almost all key respondents who were interviewed acknowledged the importance of a formal appeals mechanism in improving district-level health planning and priority setting. They felt that the public, as well as health workers, had the right to express their views on the district health plan. Some respondents remarked:

"*I think appeals mechanism is important because sometimes very important things are left out, instead insignificant things are included in the plan. But we have no idea where we can go and appeal*" (interview with a member of the user committee).

"*I wish there was a formal mechanism whereby the workers could express their views on the priorities published on the notice boards*" (interview with a health worker).

In contrast, however, the majority of those who were interviewed felt that an appeals mechanism was not feasible in their context. Pertaining to the constraints or obstacles to the appeals mechanism, respondents mentioned low-level of public awareness, lack of appeals culture, and inadequate participation of the public in the priority-setting process. The fact that funds were often earmarked for certain purposes was also seen as an obstacle to the appeals mechanism. Some members of the CHMT remarked:

"*Appeal is a very difficult phenomenon. To whom should they appeal? This thing is simply not there.... Many staff question underneath. It has never happened for staff to question things publicly. You may hear just informal complaints, but they don't have the ability to speak these out in meetings. On the part of the community, that's even more difficult*" (interview with a member of the CHMT).

"*I think currently that is not possible. I don't think anyone can appeal... How could one appeal against priorities which are the district health plan if they don't know how they were arrived at?*" (interview with a member of the CHMT).

These observations were consistent with the analysis of interviews of members of health facility committees and boards representing the community, and local government officials. The interviews indicated that there was a considerable lack of knowledge of how to appeal, which some respondents illustrated this way:

"*You cannot change things that your senior brings to you. Ideally, that is how it should be but the people lack understanding. Most of us do not understand these things so much that we may be able to question things that our leaders bring to us. We just receive most of the items, even though we are supposed to determine them ourselves*" (interview with a member of the CHSB).

"*As an ordinary worker, I am not in a position to reject anything. I cannot ask the District Medical Officer or the CHMT to explain to me why they made certain decisions. Some issues are beyond our power*" (interview head of health centre)

### Leadership and prioritisation process: signs of desired change?

The A4R framework requires that there should be organizational leadership and public regulation of the priority-setting process to ensure that the conditions of *relevance*, *publicity *and *appeals *are met. The analysis of interview data revealed that some of the CHMT members had a desire to implement the A4R conditions in priority-setting processes and in day-to-day decision-making practices. Several actions of the CHMT demonstrated its ambition to facilitate the implementation of the A4R conditions: first, the CHMT members took initiatives to write letters to the catchment areas (district hospital, health centres and dispensaries) so that they could identify their priorities and submit them to the District Medical Officer. Second, CHMT members strategically decided to visit twelve villages in order to solicit priorities from the members of the community. Attempts were also made by the CHMT to consult hospital staff about the identification of hospital priorities. Third, the CHMT members took deliberate steps to publicise district priorities on the district hospital notice board, at health facilities and in ward and village offices:

"*Beginning from last year (2009) after the completion of the district health plan we display a summary of the priorities on notice boards at the hospital, in health centres and in ward and village offices. We went even a step further by writing them letters requesting them to bring their opinions about their priorities and the allocation of resources*" (interview with a member of the CHMT).

"*We have started to stick the plan on several notice boards in hospitals, health centres and in ward offices. Even here on the notice boards you will see that we have stuck the suggested priorities*" (a member of the CHMT).

However, despite the emphasis of the planning guidelines and health policy on partnership in planning and the priority-setting process, CHMT members made little effort to involve non-health professionals in the preparation of the Comprehensive Council Health Plan. At the same time, the number of professionals involved had been increasing every year during the annual planning process.

## Discussion

This study aimed at exploring the acceptability of the A4R framework from the perspectives of CHMT members, local government officials, the health workforce, and members of user boards and committees. Increasing calls for decision-makers to be explicit about priorities (and the rationales behind them), coupled with the growing acknowledgment that priority-setting in health care is partly subjective and value-based in nature, has led to greater expectations that the A4R framework could help to improve priority-setting and resource allocation in health care institutions. Understanding the perceptions of stakeholders is crucial for the proper implementation of the A4R framework and could, in turn, help to assess the feasibility and sustainability of the innovation in priority-setting and decision making processes in the district. It is thought that this is the first study to document the actual experience of implementing the A4R framework in the planning and priority-setting process in low-income countries.

The picture of the A4R framework that emerged from the respondents was, overall, a positive one. The approach was seen as an important tool that could be used for improving priority-setting and health service delivery: first, all respondents shared the opinion that involving multiple stakeholders would ensure that a wide range of relevant values and principles would be taken into account, and thus this would improve fairness, transparency and legitimacy of the priorities identified. Second, all categories of respondents recognised that transparency had the potential to enhance the democratic process by helping the members of the community to learn how to allocate health care resources thoughtfully and fairly. Further, respondents widely shared the view that a formal appeals mechanism would provide opportunities for people to express their dissatisfaction with decisions and revisions, based on evidence. This finding resonates with the previous study of the district health planners in Tanzania by Mshana et al. [19]. Mshana *et al*. presented the framework to district health planners in a series of capacity-building workshops. Participants liked the framework, especially the extensive participation it called for, the strong expectation of transparency, and the potential for including a wider range of values.

However, in our study, a few aspects of the A4R framework were perceived as problematic by a majority of respondents. First, a majority of the CHMT members felt that adding other values and criteria was complex and easier said than done. They argued that the MoHSW had already highlighted criteria to be followed by the districts when preparing health plans. It was evident from the study that the high level of conditionality associated with the use of planning guidelines gave the CHMT, at least theoretically, little room to add other values and criteria. However, a recent study in the district documented that the planning and priority-setting was seldom evidence-based [17]. According to these authors, priority-setting usually occurred in the context of budget cycles and the process was driven by historical allocation; the use of epidemiological or cost-effectiveness evidence tended to be only a small component of the decisions. These authors also found that district health priorities were rarely implemented as planned, and lots of unstructured priority resetting happened throughout the year.

Closely related to this, concerns were also expressed by the CHMT regarding the involvement of multiple stakeholders in the planning process. The findings showed that there was fear among CHMT members of including additional stakeholders in planning and priority-setting meetings. One of the reasons provided was that many stakeholders did not have the knowledge, skills and experience to effectively contribute to priority-setting decisions. However, when CHMT members took initiatives to visit villages, to solicit community priorities, they were astonished how lay people provided useful information which was important in the preparation of the district health plan. Other studies carried out in Uganda and Tanzania have also shown that when lay people are provided with evidence they are able to engage in simulated priority-setting decisions [25,26]. Similarly, a study of priority-setting in Canada showed that service users can make a strong contribution to the process, provided that they are given time to build trust with other members of the decision making group [27]. It can, therefore, be argued that well-engaged community members could significantly contribute to non-expert values and criteria such as acceptability, feasibility, community support options and willingness, NGO partnership, age weighting etc.

Lack of funds and planning guidelines imposed by the national government were also frequently mentioned by the CHMT members as barriers to stakeholder involvement in the planning process. A review of the planning guidelines, however, shows that the CHMT enjoys a reasonable level of autonomy in terms of decision making and priority setting. The central government has delegated substantial decision making authority to the CHMT over a number of domains, including the opportunity to decide the number and type of lay stakeholders invited to the planning meetings, should the need arise. While lack of funds may continue to impinge on the CHMT's desire to broaden stakeholder participation, much depends on the willingness of the district authorities to set aside funds to this effect. Besides basket funds, which are often earmarked for specific activities, the district authority receives block grants over which local authorities can make relatively unrestricted choices. In addition, the district authorities often assume larger responsibilities for funding their services by assigning local revenues from taxes and other sources. Similarly, lack of funds might have been falsely used as an excuse against involving multiple stakeholders in planning meetings; part of this conclusion comes from the inconsistencies of the CHMT's actions during the preparation of the annual district health plans. For instance, while the CHMT claimed it had insufficient funds to invite more stakeholders to the planning meetings, including the public, the number of involved and paid for medical professionals has been increasing every year during the annual planning process.

One of the possible explanations of the CHMT's fear of broadening stakeholder participation could be due to resistance to change and the perceptions of CHMT members about the compatibility of the intervention with existing values and past experiences. Indeed, studies in other contexts have demonstrated that innovations that are perceived to be compatible with organisational norms, values and ways of working are more readily adopted [28,29]. Overall, the study results suggest that these kinds of complex innovations, which involve changes in behaviour by challenging socio-political conditions and traditions, need more time than can typically be allocated for a research project.

The last concern has to do with the *appeals/revision *condition. While stakeholders in Mbarali district widely appreciated the importance of the revision and appeals condition, they also expressed their concern about the applicability and feasibility of it. They argued that the appeals mechanism was difficult to put into action and embed into the daily routine. The challenges of achieving the appeals and revisions condition pointed out by stakeholders in the district should not be surprising, given the socio-political conditions and traditions in which the A4R framework is implemented. The previous study by Maluka et al. found that the district had no culture of appealing against decisions made by authorities [17]. According to these authors, lack of transparency of the government decision-making bodies in Tanzania, coupled with poor public awareness, seemed to be the major explanatory factors behind the lack of appeals mechanisms.

Experiences from other contexts suggest that if carried out correctly, the revision and appeals condition can close the gap between decision-makers and those affected by the policies, and engage a broader range of stakeholders in the process of deliberation [30]. However, in this study, little evidence was found to support Norman Daniel's view that even if stakeholders "do not participate in the original decision making about limits, the revision/appeals condition empowers them to play a more effective role in the larger societal deliberation about the issues and to provide wider societal oversight of the limit-setting process" [7]. Given the lack of transparency of government decision-making bodies in Tanzania, coupled with the low public awareness revealed in this study, opportunities must be provided for service users to participate collaboratively with health organisations and providers in planning, delivery, monitoring and evaluation at all levels, in a dynamic and responsive way. It is not enough for the public to merely be able to follow the prioritisation process from a distance and to appeal against decisions believed to be unfair. People have to be properly informed about priority-setting decisions in order to appeal against them, otherwise they would not even know what exactly they would be appealing against [31]. This underlines the importance of the *relevance *condition aiming for initial inclusiveness of stakeholders in the mechanism for achieving compromise. In this respect, the A4R conditions may be mutually supportive, but the strongest possible initial focus on involvement across formal and informal power differences is likely to accelerate the desired change. In a review of priority-setting in hospital operational planning in Toronto, Gibson et al. proposed a fifth condition, the 'empowerment condition', which requires that there should be efforts to minimise power differences in the decision-making context and to optimise effective stakeholder participation [32]. Significant efforts need to be made to empower the public, particularly user committees and boards as well as local civil society organisations. The effectiveness of decentralised health care planning and priority-setting is strongly influenced by the ability of the grassroots to hold service providers and local authorities accountable.

## Limitations of the study

The findings of this study should, however, be interpreted cautiously. While an effort was made to sample respondents from different levels of decision-making in the district, the sampling strategy does not allow for generalisation of the results. Even though generalisability was not the intention, the processes and views identified with the chosen methodology would most likely be reflective of many other districts in Tanzania. This study was a first step in a process of evaluation and improvement in the priority-setting process of this district. Since this study was carried out just two years after the beginning of the intervention in the district, the question of whether priority-setting in the district is legitimate or fair has not been examined. However, the study contributes to our understanding of the acceptability of the A4R framework in improving planning and priority-setting processes in low-income countries.

## Conclusion

This study was one of the first assessments of the applicability of Accountability for Reasonableness in health care priority-setting in Tanzania. Specifically, the study aimed at exploring the acceptability of the A4R framework from the perspectives of the CHMT members, local government officials, health workforce and members of the user committees and boards. The analysis suggests that the Accountability for Reasonableness framework may be a useful tool for improving the priority-setting process in the context of poor resource settings.

The results of this study have three important implications for the implementation of a fair and legitimate priority-setting process: firstly, the findings imply that attempts to establish fair priority-setting mechanisms have to recognise constraints in the local contexts of socio-political conditions and traditions; the desired change is unlikely to come about without direct attention paid to these. Thus, the A4R framework should be implemented with flexibility to allow for the adjustments to the local contextual issues as described above. Secondly, given the low level of public awareness, and the novelty of some aspects of the A4R approach, the implementation of the A4R conditions would be strengthened through capacity-building of all relevant stakeholders. Thirdly, more work should be done to explore appropriate ways of reviewing priority-setting decisions and addressing disagreements constructively. Such a mechanism would help improve the quality of decisions by providing opportunities for new information to be brought forward. Similarly, the process would help realise the key ethical concept of responsiveness.

## List of abbreviations

**A4R**: Accountability for Reasonableness; **ART**: Action Research Team; **CCHP**: Comprehensive Council Health Plan; **CHMT**: Council Health Management Team; **CHSB**: Council Health Service Board; **DED**: District Executive Director; **EHP**: Essential Health package; **FBOs**: Faith Based Organizations; **MoHSW**: Ministry of Health and Social Welfare; **NGOs**: Non governmental Organizations; **PMO-RALG**: Prime Minister's Office Regional Administration and Local Government; **REACT**: Response to Accountable Priority Setting for Trust in Health Systems;

## Competing interests

The authors declare that they have no competing interests.

## Authors' contributions

All five authors contributed to the original design of the study. SM carried out the data collection. SM, AKH and JB analysed the data. SM drafted the manuscript and all authors contributed to the revising of this manuscript. All authors read and approved the final manuscript.

## Appendix 1: Interview guide for CHMT members

### Process of setting priorities

1. In reality, who is involved in the priority-setting process? Who else should have been involved? In what capacity?

2. What factors/values or criteria are taken into account when identifying priorities? Who decides on the factors to be taken into account?

3. Which other factors do you think should have been used in setting priorities?

4. What is your opinion on involving other stakeholders, including community, in the priority-setting process?

### Transparency & Publicity

5. Do you think the priority-setting process in your district is transparent? To what extent and why?

6. Do you think the priority-setting process, and the reasons behind the decisions taken, are widely publicised to the public and relevant stakeholders? Please explain.

7. In your opinion, do you think it is important for the CHMT to publicise priorities of the district and to justify why those priorities were selected? What are the advantages and problems of doing this?

8. What are the problems of institutionalising a transparent priority-setting process in the district?

### Appeals and revision

9. How do you resolve disagreements on priority?

10. Are you able to appeal and ask for revisions if the priorities are not relevant to your values and needs?

11. Do you think it is important for the district to have formal mechanisms for stakeholders, including the public, to appeal in case they are not satisfied with the priorities of the district? Have there been any problems with this?

### Overall view on the priority-petting process

12. In your opinion, and thinking specifically in comparison to previous years, what were the strengths of the priority-setting process in the 2009/2010 planning year¬

13. What were the key weaknesses of the priority-setting process in the 2009/2010 planning year?

14. What changes have you seen with regard to the priority-setting process over the last two years?

15. What could be done to improve the priority-setting process in the district?

## Pre-publication history

The pre-publication history for this paper can be accessed here:

http://www.biomedcentral.com/1472-6963/10/322/prepub
